# Inhibitory activity of meso-dimercaptosuccinic acid against IMP metallo-*β*-lactamase variants in *Pseudomonas aeruginosa*

**DOI:** 10.1093/femsle/fnaf052

**Published:** 2025-05-28

**Authors:** Jacqueline Findlay, Hector Moreno, Gilbert Greub, Christophe Le Terrier, Maxime Bouvier, Patrice Nordmann

**Affiliations:** Medical and Molecular Microbiology, Faculty of Science and Medicine, University of Fribourg, 1700 Fribourg, Switzerland; Medical and Molecular Microbiology, Faculty of Science and Medicine, University of Fribourg, 1700 Fribourg, Switzerland; Institute of Microbiology, University Hospital Centre and University of Lausanne, 1011 Lausanne, Switzerland; Institute of Microbiology, University Hospital Centre and University of Lausanne, 1011 Lausanne, Switzerland; Medical and Molecular Microbiology, Faculty of Science and Medicine, University of Fribourg, 1700 Fribourg, Switzerland; Geneva University Hospitals, Division of Intensive Care, Department of Acute Care Medicine, 1205 Geneva, Switzerland; Department of Anaesthesiology, Pharmacology, Intensive Care and Emergency Medicine, Faculty of Medicine, University of Geneva, 1211 Geneva, Switzerland; Swiss National Reference Center for Emerging Antibiotic Resistance (NARA), University of Fribourg, 1700 Fribourg, Switzerland; Medical and Molecular Microbiology, Faculty of Science and Medicine, University of Fribourg, 1700 Fribourg, Switzerland; Swiss National Reference Center for Emerging Antibiotic Resistance (NARA), University of Fribourg, 1700 Fribourg, Switzerland

**Keywords:** *Pseudomonas aeruginosa*, carbapenemase, MBL, IMP, inhibitor, beta-lactams

## Abstract

The treatment of infections caused by MBL-producing *Pseudomonas aeruginosa*, including IMP-producers, can be challenging since MBLs confer resistance to most clinically available ß-lactams, leaving few viable options. Thiol-containing compounds, such as meso-dimercaptosuccinic acid (DMSA), have been suggested as inhibitors of MBLs, and particularly have been proposed to exert effective activity against IMP-1 enzymes. In this context, we sought to determine the activity of DMSA, in combination with carbapenems or cephalosporins, against diverse MBL and carbapenemase variants, particularly focussing on IMP variants. In recombinant strains producing either IMP-1, IMP-10, or IMP-13, both with and without a functional OprD, synergy was observed with ceftazidime (CAZ), cefepime (FEP), and meropenem. Negligible changes were observed for other MBLs and no effect was observed in stains producing KPC-2. Testing of the CAZ/DMSA and FEP/DMSA combinations against clinical IMP-producing *P. aeruginosa* isolates resulted in fractional inhibitory concentration indexes indicating synergy. Enzymatic assays showed a significantly increased inhibitory activity of DMSA against the IMP variants, compared to the other enzymes tested. These findings highlight a possible role of DMSA or DMSA-like compounds to be developed for the treatment of infections caused by IMP-producing *P. aeruginosa*.

## Teaser

This study highlights a possible role of DMSA or DMSA-like compounds to be developed for the treatment of infections caused by IMP-producing *Pseudomonasaeruginosa*.

## Introduction

Carbapenem-resistant *Pseudomonas aeruginosa* (CRPA) represent an imminent threat to public health and are listed as “high” priority pathogens by the World Health Organisation (WHO 2024). Carbapenem resistance in CRPA is predominantly due to permeability defects, specifically porin OprD loss/modifications, and/or the production of acquired carbapenemases, mostly metallo-ß-lactamases (MBLs), including VIM-, NDM-, and IMP-type enzymes (Hong et al. 2015, Reyes et al. 2023). IMP-type carbapenemaes were first described in a CRPA isolate in Japan in 1991 (Watanabe et al. 1991) and have since gone on to become one of the most frequently reported MBLs in CRPA, particularly in Eastern Asia (Horcajada et al. 2019). To date, 111 IMP enzyme variants have been identified (http://www.bldb.eu), mostly reported in Enterobacterales, *P. aeruginosa* and, to a lesser extent, *Acinetobacter baumannii* (Arer and Kar 2023). The treatment of infections caused by MBL-producing *P. aeruginosa*, including IMP-producers, can be challenging since MBLs confer resistance to most clinically available ß-lactams, leaving few viable options (Hong et al. 2015, Horcajada et al. 2019, Reyes et al. 2023). In recent years, several novel ß-lactam/ß-lactamase inhibitor combinations have been developed for the treatment of MBL-producers in Enterobacteriaceae, including aztreonam-avibactam, cefepime-taniborbactam, and meropenem-xeruborbactam—all of which are not yet approved but are currently undergoing clinical trials (Arer and Kar 2023). However, taniborbactam (TAN) has been shown to exhibit poor activity against IMP-producers (Hamrick et al. 2020, Le Terrier et al. 2024) and xeruborbactam (XER) was reported to exhibit limited activity against IMP-10, which differs from IMP-1 by single amino acid, Val67Phe (Le Terrier et al. 2024). An analysis of the currently known 111 IMP enzyme variants showed that 21/111 (19%) harbour this Val67Phe change, relative to IMP-1. The thiol-containing compound, meso-dimercaptosuccinic acid (DMSA), is a heavy metal chelator which has been approved for over 30 years for the treatment of lead and mercury intoxication (Bradberry et al. 2009). Thiol-containing compounds have been suggested as inhibitors of MBLs and particularly have been proposed to exert effective activity against IMP-1 enzymes (Hammond et al. 1999, Toney et al. 2001). MBLs contain zinc ions at their active site, which are a key determinant for their *β*-lactamase activity, and previously, it has been shown that DMSA at 3 mM can significantly reduce the carbapenem [imipenem (IPM) and meropenem (MEM)] MICs of MBL-producers in isogenic strains of *P. aeruginosa* (Bouvier et al. 2025). This effect was shown to be mediated via an additive, rather than synergistic, interaction (Bouvier et al. 2025). In this context, we sought to determine the activity of DMSA, in combination with carbapenems and cephalosporins, against diverse MBL and carbapenemase variants, but particularly focussing on IMP variants.

## Materials and methods

### Bacterial strains

*Pseudomonas aeruginosa* isolate, PAO1, was used alongside its isogenic counterpart, PAO1 Tn*oprD*, which lacks a functional OprD porin (Held et al. 2012). *β*-lactamases, *bla*_IMP-1_, *bla*_IMP-10_, *bla*_IMP-13_, *bla*_NDM-1_, *bla*_SPM-1_, *bla*_VIM-1_, *bla*_VIM-2_, and *bla*_KPC-2_, had previously been cloned into the pUCP24 shuttle vector (Bouvier et al. 2025), and were transformed by electroporation into both PAO1 and PAO1 Tn*oprD*.

Thirty-two carbapenem-resistant clinical *P. aeruginosa*, with previously defined carbapenem-resistance mechanisms, were included. These comprised of MBL-producers including 18 IMP-producers [IMP-1 (*n* = 7), IMP-7 (*n* = 5), IMP-13 (*n* = 3), IMP-4, IMP-10, IMP-18], and isolates producing NDM-1 (*n* = 2), VIM-2 (*n* = 2), VIM-4 (*n* = 2), VIM-36 (*n* = 2), one isolate that co-produced IMP-4, OXA-181, and VIM-2, and lastly, one isolate that co-produced both NDM-1 and VIM-2. In addition, five non-MBL-producing carbapenem-resistant isolates were also included that comprised producers of KPC-2 (*n* = 2), GES-5 and OXA-181 (*n* = 1), and non-carbapenemase-producing isolates with permeability (OprD) defects (*n* = 2).

### Susceptibility testing and checkerboard assays

Minimal inhibitory concentrations (MICs) to DMSA alone were determined against all isogenic strains expressing different carbapenemases and against clinical isolates, in 100 mg/l increments (100–1200 mg/l range). MICs of ceftazidime (CAZ), cefepime (FEP), IPM, MEM, alone and in checkerboard assays with DMSA at different concentrations (100, 200, and 300 mg/l) were determined by broth microdilution for all isogenic strains. MICs of DMSA, CAZ, FEP and checkerboards for the combinations, CAZ/DMSA and FEP/DMSA, were performed for all clinical isolates. All susceptibility testing results were interpreted according to CLSI guidelines (CLSI 2023). The fractional inhibitory concentration index (FICI) was calculated and interpreted as follows: the MIC of drug A in combination/the MIC of drug A alone + the MIC of drug B in combination/the MIC drug B alone. FICI ≤ 0.5 = synergy; 0.5 < FICI ≤ 1 = additive; FICI 1 < FICI < 2 = indifference, and FICI ≥ 2 = antagonism. All susceptibility testing/checkerboard assays were performed in triplicate.

### Determination of 50% inhibitory concentration (IC_50_)

IC_50_ measurements were performed as previously described (Mueller et al. 2019). Briefly, *β*-lactamases from crude extracts were prepared and used for the measurement of the specific activity in 100 mM sodium phosphate buffer (pH 7.0). Measurements were performed in a Genesys 10S UV/VIS spectrophotometer (Thermo Scientific) spectrophotometer using a wavelength of 262 nm for cephalothin. The 50% inhibitory concentration (IC_50_) for each carbapenemase variant was determined as the concentration of DMSA, TAN, or XER, which reduced the hydrolysis rate of 100 μM cephalothin (CEF) by 50%. Extracts were preincubated with DMSA for 3 min prior to the addition of CEF and all measurements were performed in triplicate.

## Results and discussion

### Synergy testing in isogenic strains

MICs for DMSA alone were performed against isogenic strains PAO1 and PAO1 Tn*oprD* producing one of eight different carbapenemases; IMP-1, IMP-10, IMP-13, NDM-1, SPM-1, VIM-1, VIM-2, and KPC-2 (Table 1). All strains exhibited an MIC to DMSA alone of 900 mg/l. Checkerboard assays were then performed for all strains using DMSA at 100, 200, and 300 mg/l, in combination with either CAZ, FEP, IPM, or MEM. In isolates producing either IMP-1, IMP-10, or IMP-13 both with and without a functional OprD, synergy was observed to CAZ and FEP at all three DMSA concentrations, and to MEM at DMSA concentrations of 200 and 300 mg/l, as indicated by the FICIs (Table 1 and Table S1). However, in strains expressing NDM-1, SPM-1, VIM-1, VIM-2, and KPC-2 carbapenemases, synergy was only observed in the SPM-1 and VIM-1 strains to CAZ (DMSA 200 mg/l) and SPM-1 to MEM (DMSA 200 and 300 mg/l). Additive or indifferent FICIs were observed for most other strains, regardless of the antibiotic combination and DMSA concentration, indicating an IMP-specific effect. The IPM/DMSA combination produced almost no synergistic FICI values. The lack of a functional OprD was shown to significantly affect the IPM/DMSA combination. This could be expected since it is well known that OprD acts as a preferred entry pathway for IPM into the *P. aeruginosa* cell but does not affect the permeation of cephalosporins and only slightly affects meropenem (Huang and Hancock 1993).

**Table 1.** MICs and FICIs of recombinant strains PAO1 and PAO1 Tn*oprD* producing different carbapenemases with DMSA at 100 mg/l.

Strain	FEP	FEP/DMSA	FICI	CAZ	CAZ/DMSA	FICI	IPM	IPM/DMSA	FICI	MEM	MEM/DMSA	FICI
IMP-1	512	64	**0.24**	1024	256	**0.36**	16	8	0.61	64	32	0.61
IMP-1 Tn*oprD*	512	64	**0.24**	1024	256	**0.36**	64	64	1.11	128	64	0.61
IMP-10	256	64	**0.36**	2048	256	**0.24**	8	4	0.61	128	32	**0.36**
IMP-10 Tn*oprD*	256	64	**0.36**	2048	256	**0.24**	64	32	0.61	256	64	**0.36**
IMP-13	16	1	**0.17**	64	4	**0.17**	1	1	1.11	4	1	**0.36**
IMP-13 Tn*oprD*	16	1	**0.17**	64	4	**0.17**	8	8	1.11	8	2	**0.36**
NDM-1	256	256	1.11	4096	4096	1.11	32	32	1.11	128	64	0.61
NDM-1 Tn*oprD*	256	256	1.11	4096	4096	1.11	128	128	1.11	256	128	0.61
SPM-1	64	64	1.11	512	256	0.61	2	2	1.11	32	16	0.61
SPM-1 Tn*oprD*	64	64	1.11	512	256	0.61	16	16	1.11	64	32	0.61
VIM-1	512	256	0.61	2048	1024	0.61	64	64	1.11	64	64	1.11
VIM-1 Tn*oprD*	512	256	0.61	2048	1024	0.61	128	128	1.11	128	128	1.11
VIM-2	2	1	0.61	8	4	0.61	4	4	1.11	4	4	1.11
VIM-2 Tn*oprD*	2	2	1.11	8	4	0.61	32	32	1.11	8	8	1.11
KPC-2	512	256	0.61	128	128	1.11	128	128	1.11	256	256	1.11
KPC-2 Tn*oprD*	512	512	1.11	128	128	1.11	512	512	1.11	512	512	1.11

Numbers in bold illustrate synergistic FICIs (≤0.5).

### Synergy testing in clinical isolates

We sought to assess whether DMSA in combination with CAZ or FEP, could be a viable treatment option in clinical isolates and evade the effects of permeability loss that are typically observed in CRPA (Hong et al. 2015). MIC testing was performed using DMSA at fixed concentrations of 100, 200, and 300 mg/l in combination with CAZ and FEP, and with CAZ or FEP alone. Thirty-two clinical CRPA isolates, with previously characterized carbapenem-resistance mechanisms, were tested. Most (18/32) of the clinical isolates produced a single IMP carbapenemase variant and in total, producers of six different IMP variants (IMP-1, -4, -7, -10, -13, -18) were assessed. Overall, the CAZ/DMSA and FEP/DMSA combinations exhibited the greatest activity against IMP-producers, with synergistic FICIs observed at all three DMSA concentrations (Table 2). Only additive or indifferent FICIs were observed for strains producing other MBLs, producing KPC-2, or with permeability defects (OprD loss) only. Despite the high MICs to FEP only (64–1024 mg/l) in the 18 IMP-producers, two (DMSA at 100 mg/l), 6 (DMSA at 200 mg/l) and 10 (DMSA at 300 mg/l) strains exhibited MICs that rendered them sensitive according to CLSI guidelines (CLSI 2023).

**Table 2.** Characteristics and MICs of the 32 clinical isolates in this study.

		MICs (mg/l) and FICI															
Isolate	Carbapenem resistance mechanism	DMSA	CAZ	CAZ/DMSA100	FICI	CAZ/DMSA200	FICI	CAZ/DMSA300	FICI	FEP	FEP/DMSA100	FICI	FEP/DMSA200	FICI	FEP	FICI	
D1	IMP-1	600	1024	128	**0.29**	64	**0.40**	32	0.53	512	64	**0.29**	64	**0.46**	16	0.53	
D2	IMP-1	600	1024	256	**0.42**	64	**0.40**	64	0.56	256	64	**0.42**	32	**0.46**	16	0.56	
D3	IMP-1	700	512	64	**0.27**	32	**0.35**	16	**0.46**	256	64	**0.39**	32	**0.41**	32	0.55	
D4	IMP-1	900	1024	128	**0.24**	64	**0.28**	32	**0.36**	256	32	**0.24**	32	**0.35**	16	**0.40**	
D5	IMP-1	800	512	128	**0.38**	32	**0.31**	16	**0.41**	256	16	**0.19**	16	**0.31**	8	**0.41**	
D6	IMP-1	800	512	64	**0.25**	32	**0.31**	32	**0.44**	256	32	**0.25**	8	**0.28**	4	**0.39**	
D7	IMP-1	800	1024	256	**0.38**	128	**0.38**	128	**0.50**	512	128	**0.38**	32	**0.31**	32	**0.44**	
D8	IMP-4	500	2048	512	**0.45**	256	0.53	128	0.66	1024	64	**0.26**	32	**0.43**	16	0.62	
D9	IMP-7	700	256	32	**0.27**	16	**0.35**	8	**0.46**	64	8	**0.27**	4	**0.35**	4	**0.49**	
D10	IMP-7	600	512	64	**0.29**	16	**0.36**	16	0.53	256	32	**0.29**	16	**0.40**	16	0.56	
D11	IMP-7	600	512	128	**0.42**	32	**0.40**	16	0.53	128	16	**0.29**	8	**0.40**	8	0.56	
D12	IMP-7	600	512	64	**0.29**	32	**0.40**	16	0.53	128	16	**0.29**	32	0.58	4	0.53	
D13	IMP-7	700	512	64	**0.27**	32	**0.35**	32	**0.49**	128	16	**0.27**	16	**0.41**	8	**0.49**	
D14	IMP-10	900	1024	128	**0.24**	64	**0.28**	32	**0.36**	512	64	**0.24**	64	**0.35**	64	**0.46**	
D15	IMP-13	500	512	32	**0.26**	16	**0.43**	8	0.62	128	16	**0.33**	8	**0.46**	8	0.66	
D16	IMP-13	600	512	64	**0.29**	16	**0.36**	8	0.52	128	8	**0.23**	4	**0.36**	2	0.52	
D17	IMP-13	700	256	64	**0.39**	16	**0.35**	16	**0.49**	128	16	**0.27**	4	**0.32**	4	**0.46**	
D18	IMP-18	600	1024	128	**0.29**	64	**0.40**	32	0.53	256	32	**0.29**	16	**0.40**	8	0.53	
D19	IMP-4, OXA-181, VIM-5	600	4096	4096	1.17	4096	1.33	4096	1.50	512	512	1.17	512	1.33	512	1.50	
D20	NDM-1	700	4096	4096	1.14	4096	1.29	4096	1.43	512	512	1.14	256	1.29	256	0.93	
D21	NDM-1	700	4096	4096	1.14	4096	1.29	4096	1.43	1024	1024	1.14	1024	1.29	512	0.93	
D22	NDM-1, VIM-2	700	4096	4096	1.14	2048	1.29	2048	0.93	128	128	1.14	128	1.29	128	1.43	
D23	VIM-2	700	32	32	1.14	32	1.29	32	1.43	16	8	0.64	8	0.79	8	0.93	
D24	VIM-2	600	32	16	0.67	16	0.83	16	1.00	8	8	1.17	4	0.83	4	1.00	
D25	VIM-4	600	32	16	0.67	16	0.83	8	0.75	32	16	0.67	16	0.83	8	0.75	
D26	VIM-4	700	128	64	0.64	64	0.79	32	0.68	64	64	1.14	32	0.79	16	0.68	
D27	VIM-36	800	512	512	1.13	256	0.75	256	0.88	32	16	0.63	16	0.75	16	0.88	
D28	GES-5, OXA-181	600	16	16	1.17	16	1.33	8	1.00	8	8	1.17	4	1.33	8	1.50	
D29	KPC-2	800	16	16	1.13	16	1.25	16	1.38	64	64	1.13	64	1.25	64	1.38	
D30	KPC-2	800	64	64	1.13	64	1.25	32	0.88	512	512	1.13	512	1.25	256	0.88	
D31	Permeability	1000	128	128	1.10	128	1.20	64	0.80	32	32	1.10	32	1.20	8	0.55	
D32	Permeability	700	16	16	1.14	16	1.29	16	1.43	4	8	1.14	4	1.29	8	1.43	

DMSA was tested at fixed concentrations of 100, 200, and 300 mg/l. Numbers in bold illustrate synergistic FICIs (≤0.5).

### Enzymatic activity

Enzymatic assays showed that the inhibitory activity of DMSA was considerably higher against the three IMP variants compared to the other carbapenemases (Table 3), with IC_50_ values increasing ≥3-fold, thus explaining the synergy and clinical MICs observed. The highest IC_50_ value was observed for KPC-2, at 6.93 mM (data not shown), at least 4-fold higher than that observed for any other MBLs, suggesting that the activity of DMSA is greater against MBLs compared to the non-MBL carbapenemase. However, the exact mechanism of DMSA inhibition remains to be determined. TAN gave lower IC_50_ values against all enzymes except against the IMP-expressing strains in which TAN is known to exhibit poor activity. XER generally exhibited lower IC_50_ values against all enzymes except IMP-10, to which the DMSA had a slightly lower IC_50_ (50 µM for DMSA vs 73 µM for XER). IMP-10 is a single amino variant of IMP-1 (Val67Phe) and XER is known to have limited activity against this variant (Le Terrier et al. 2024).

**Table 3.** Inhibitory concentrations of DMSA, TAN, and XER against IMP-1, IMP-10, IMP-13, VIM-1, VIM-2, NDM-1, SPM-1, and KPC-2.

	IC50 (µM)		
Enzyme	DMSA	TAN	XER
IMP-1	120	>1000	2.7
IMP-10	50	>1000	73
IMP-13	350	>1000	3.6
NDM-1	>1000	3.0	4.3
SPM-1	>1000	2.7	>1000
VIM-1	>1000	0.8	0.5
VIM-2	>1000	0.5	0.1
KPC-2	>1000	ND	ND

ND; not determined.

## Conclusion

The repurposing of “old” drugs with known safety records presents an attractive prospect for the identification of new antimicrobial inhibitors, potentially allowing the rejuvenation of antimicrobials rendered ineffective by resistance. Here, we showed that the addition of the heavy metal chelator, DMSA, to both CAZ and FEP was effective in significantly reducing MICs in IMP-producing *P. aeruginosa* strains. Of note, DMSA may cover the window of inefficacy of TAN that is ineffective against IMP producers. Previous studies in humans have reported a wide range of achievable plasma and urine concentrations of DMSA with peaks ranging from ∼20 to ∼60 µM in plasma and ∼150 to ∼1000 µmol in studies based upon single 10 mg/kg dosing (Aposhian et al. 1989, Maiorino et al. 1989, Maiorino et al. 1990, Dart et al. 1994, van Eijkeren et al. 2016). The effective concentration used in this study, 100 mg/l, equates to 548 µM, which is relatively high, however DMSA is known to be well tolerated at a 30 mg/kg dose and is usually administered over a long period, ranging from 5 to 26 days (van Eijkeren et al. 2016). Additionally, DMSA has previously been shown to be effective and well-tolerated at high concentrations in a mouse peritonitis model of infection with MBL-producing in *E. coli* and subsequently (Cheminet et al. 2020), further studies in an animal infection model with *P. aeruginosa* will be necessary to determine if higher urine and plasma concentrations are achievable. Overall, these findings highlight a possible role of DMSA or DMSA-like compounds for the treatment of infections caused by IMP-producing *P. aeruginosa*.
